# Enrichment Preferences of FIV-Infected and Uninfected Laboratory-Housed Cats

**DOI:** 10.3390/v10070353

**Published:** 2018-07-03

**Authors:** Claudia J. Kennedy, Andrea E. Thomson, Emily H. Griffith, Jonathan Fogle, B. Duncan X. Lascelles, Rick B. Meeker, Barbara L. Sherman, Margaret E. Gruen

**Affiliations:** 1 Department of Animal Science, College of Agriculture and Life Sciences, North Carolina State University, Raleigh, NC 27695, USA; cjkenne5@ncsu.edu; 2 Department of Clinical Sciences, North Carolina State College of Veterinary Medicine, Raleigh, NC 27606, USA; andrea_thomson@ncsu.edu (A.E.T.); dxlascel@ncsu.edu (B.D.X.L.); barbara_sherman@ncsu.edu (B.L.S.); 3 Department of Statistics, College of Sciences, North Carolina State University, Raleigh, NC 27695, USA; eghohmei@ncsu.edu; 4 Department of Population Health and Pathobiology, North Carolina State College of Veterinary Medicine, Raleigh, NC 27606, USA; jefogle@ncsu.edu; 5 Department of Neurology, University of North Carolina at Chapel Hill, Chapel Hill, NC 27599, USA; meekerr@neurology.unc.edu

**Keywords:** FIV, enrichment, welfare, feline, laboratory animal

## Abstract

Environmental enrichment is critical for alleviating stress in laboratory felines. However, there is a paucity of information about suitable enrichment for cats. This study aimed to determine preferred enrichment options of individually-housed, castrated male domestic short hair cats (*Felis catus*) used in a longitudinal study of the effects of chronic feline immunodeficiency virus (FIV) infection, and to determine if the FIV status of the cats affected enrichment preferences. Preference testing was performed with two types of grooming brushes, three different interactive play options, including a laser, ball, and petting interaction with a familiar investigator, and two types of toenail conditioning objects. We found that cats elected to be brushed, preferred social interaction and play with the laser to the ball, and preferred to scratch on an inclined-box toenail conditioning object compared to a horizontal, circular toenail conditioning object. There were individual preferences for enrichment opportunities. There were no differences in preferences between FIV-infected and sham-infected cats. These enrichment preferences may be used to advise laboratory animal facilities and researchers about how to best accommodate the behavioral needs of laboratory cats.

## 1. Introduction

In laboratory animal facilities, efforts are made by researchers, veterinarians, and caretakers to enrich the lives of animals in captivity beyond basic biological needs, such as housing, food, water, and resting sites. Indeed, many laboratory animal facilities are mandated to provide environmental enrichment for residents [1]. Environmental enrichment provides enhancements in order to meet animals’ behavioral needs, providing stimulation and reducing undesirable behaviors and stress [2]. The goal of enrichment is to allow animals to express natural species-specific behaviors, such as running, jumping, climbing, play, predatory simulation (when appropriate), and positive social interactions in order to reduce stress [3] and improve their overall well-being [4]. Enrichment comes in various forms, including contact with conspecifics, toys, human interaction, caging type and furnishings, and auditory or olfactory stimulation [4,5]. In laboratory animals, enrichment must conform to facility requirements for cleaning or disposal in order to prevent enrichment items serving as fomites for infectious diseases.

Enrichment recommendations for cats in laboratory settings are similar to those made to meet the needs of cats in their homes [6]. In the physical environment, requirements include adequate space for the cat to move, structures for the cat to interact with, resting places, hiding places, and stimuli in order to meet the needs of the cat for exercise, activity, rest, and coping behaviors. Within the social environment, enrichment focuses on positive interactions with conspecifics and human caregivers [5]. Other suggested welfare needs of cats include scratching substrates for nail conditioning and scent deposition, and an assortment of toys for play and object manipulation. While these welfare and enrichment recommendations have been evaluated for domestic housecats [4], they have not been systematically evaluated for laboratory-housed cats.

There is evidence that providing suitable enrichment may have effects on neurochemistry and resulting behavior, thereby improving data quality [7]. In addition to meeting recommendations for best care, environmental enrichment can attenuate many stressors that exist in a laboratory environment [8]. Procedures that require restraint, those that produce some level of discomfort, and the approach and presence of an unfamiliar person may contribute to laboratory stress and can negatively impact the welfare of cats housed in laboratory animal facilities. With feline models, stress may be reflected in elevated cortisol levels, diminished appetite, elective social isolation or aggression, suboptimal grooming behavior, and increased tendency to hide [9]. Animals in stressful environments and those unable to express their natural behaviors can impact “baseline” laboratory and behavioral data. These outcomes can compromise the quality of the research data [10].

While there are recommendations available for meeting the needs of confined cats [5,6,11], very little research exists on the actual preferences of laboratory cats for specific types of enrichment, or how to assess the enrichment preferences of individual cats. What we, as humans, perceive to be enriching may not be what the animal actually prefers, and there may be individual differences in enrichment preferences. Preference testing is a way to “ask the animal” what it prefers or finds interactive [12]. Preference testing is typically performed by providing an animal with a choice between two or more options, while the strength of preferences has been assessed by quantifying the amount of “work” that an animal will do to access a resource ([13], and see [14] for review). Enrichment is especially important in animals maintained for long-term studies, such as studies of cats infected with feline immunodeficiency virus (FIV) [15]. Cats infected with FIV serve as a useful animal model for the study of human immunodeficiency virus (HIV) [16,17], particularly psychomotor deficits seen with long-term infection. Cats infected with FIV for 12 or more months have been shown to be hyperactive in open field testing, more easily distracted, and more likely to have errors in completing new operant tasks compared to control cats [18]. Because cats can transmit FIV via bites, FIV-infected cats are housed individually (with visual access to conspecifics). This requirement for social isolation makes provision of adequate and preferred enrichment for individual cats even more crucial.

The goals of this study were, therefore, to assess the preferences for type of enrichment in laboratory-housed cats, and to determine if FIV status impacts these preferences. We predicted that individual cats do have a preference for specific types of enrichment, and that the FIV status of the cat will not systematically impact their preferences. In presenting enrichment types, we used the traditional method of presenting two choices simultaneously for some of our enrichment types; for others, we used an intensity of interaction as a surrogate for preference. Our findings supported our predictions; we found that cats had preferences for certain enrichment options, and that there were individual preferences for enrichment types. There were no differences in preferences between FIV-infected and sham-infected cats.

## 2. Materials and Methods

All protocols described in this study were approved by the North Carolina State University Institutional Animal Care and Use Committee (Protocol 12-176-B, 28 January 2013).

### 2.1. Experimental Animals

The subjects were purpose-bred, specific-pathogen-free (SPF), castrated male domestic short hair cats (*Felis catus*) between 2–2.5 years of age and obtained from a commercial breeding facility. The cats were participating in a longitudinal study of the effects of FIV infection on physiological parameters and cognitive-motor skills (described in [19]). Cats had been FIV-infected or sham-infected via intra-cranial inoculation 9 to 13 months prior to the initiation of the present study. The FIV status of each cat had been subsequently confirmed. Although all cats participated in a series of reward-based cognitive-motor tests on a prescribed schedule, the enrichment preference testing described here was conducted during a time when cats were not undergoing cognitive-motor testing. On physical examination, all cats were in good general health. Animal technicians and behavior team members were masked to the FIV status of the cats.

### 2.2. Housing and Husbandry

Cats were housed in individual pens (188 cm high, 147 cm deep, 91 cm wide), each of which included an elevated perch, a hide, a litter pan (cleaned daily) and a water bowl. There were 5–6 pens located in each of four separate, identical rooms in the North Carolina State University Laboratory Animal Resources Unit (LAR). Cats were maintained on a 12/12-h light/dark cycle and fed a measured balanced feline dry ration (Hills Feline Hairball Control Diet, Hill’s Pet Nutrition, Inc., Topeka, KS, USA) twice daily and weighed monthly in order to maintain body condition scores of 4–5/9, as referenced on a standard body condition score chart (Purina Body Condition Score Index, http://www.purina.com/cat/weight-control/bodycondition.aspx). All cats were conditioned to handling and carrier transport [20], and were observed twice daily.

At the start of the study, minimal untested environmental enrichment protocols were in place. Each cat was provided in its pen with a feline-appropriate play toy, which was rotated weekly. For 10 min each day, animal care technicians allowed each cat out of its pen individually for exercise, nail conditioning, and exploration. Twice a week, each cat was brushed by familiar animal technicians for examination, body care and to decrease the incidence of hairballs. Periodically, cats were taken to other rooms in carriers for physical evaluation, physiological assessment, or individual behavioral testing. Each cat room was continuously provisioned with a feline pheromone diffuser (Feliway^®^ Diffuser Ceva Animal Health, LLC, Lenexa, KS, USA), purported to reduce stress in cats [21].

### 2.3. Pre-Test Period

During the pre-test period, two months prior to data collection, interviews were conducted with animal technicians in order to learn which brushes, play toys, and nail-conditioning items already approved for safety and infectious disease control by LAR were utilized by the cats. After a literature search and discussion with technicians, specific items were chosen for their prior interest from the cats, their ease of cleaning, and their cost effectiveness. One month prior to data collection, the investigator (CK) acclimated the cats to her presence by entering each cat room for 30 min twice weekly and spending approximately 5 min brushing, playing, and individually interacting with each cat in the room. Commercially available cat treats were used to lure each cat back to his pen after the interactions.

### 2.4. Testing

After the pre-test period, testing began. To evaluate behavioral choices for environmental enrichment, the cats were provided with five enrichment opportunities consisting of (1) choice of grooming brushes; (2) play with a laser light; (3) play with a 5 cm diameter ball; (4) interaction with the familiar investigator; and (5) two types of corrugated cardboard toenail conditioning objects. Each room had its own set of enrichment items, which were shared among the cats in the room, but not transferred between rooms. The order of presentation of enrichment opportunities 2–4, testing order of rooms, and testing order of cats within a room were randomized. All cats in a room were tested before moving to the next room.

Each cat was individually tested a total of six times, using a standardized protocol consisting of five enrichment opportunities. A typical test protocol is described as follows: The investigator (CK) released each cat from its pen, then kneeled on the floor approximately 1 m from the pen door. Then, the cat was offered two brush types and its choice was recorded. Next, the cat was given 1 min each to interact with a laser light, directed by the investigator, a ball, and a familiar human (the investigator) (described in detail under Enrichment Choices). If the cat tried to interact with the investigator during laser light or ball testing periods, the investigator ignored the cat. The intensity of each cat’s response was measured on a 0 to 2 activity scale. Zero (0) reflected no interest, in which the cat either did not interact with the object or did so only one time; one (1) reflected moderate interest, in which the cat interacted with the object multiple times, but lost interest throughout the trial period and wandered away; two (2) reflected intense interest, in which the cat interacted with the object during the entire trial period. At the end of each session, the cat was returned to his kennel. There, in the restricted space of the pen, he was offered two toenail conditioning objects for one minute and his choice was noted.

### 2.5. Experimental Procedures

All cats in a group were tested sequentially between the hours of 13:00 and 16:00. The trials were conducted over a period of 7 weeks when the cats were not scheduled for physiological sampling or cognitive-motor test protocols. 

### 2.6. Enrichment Choices

#### 2.6.1. Brushes

The cat was visually presented with two brushes, equidistant from the cat and held in the investigator’s hands. Brush types were a 7-row Brigitte hair brush (Brigitte Brush, Brigitte’s Brushes Company, Lanoka Harbor, NJ, USA) and a glove brush (Love Gloves, Four Paws Company, Neptune, NJ, USA). The cat made a selection by nudging or rubbing against one of the brushes, soliciting its use. The investigator recorded “no preference” if the cat did not allow interaction with either brush, and recorded “both brushes” if the cat alternated between brushes. The investigator was careful to not interact with the cat in other ways besides brushing, as to eliminate the cat choosing to be brushed based on human interaction.

#### 2.6.2. Laser

During the laser task, the investigator turned on a handheld laser light designed to stimulate chasing activity in cats (Fun Beam Interactive Laser Toy, Petlinks System, San Rafael, CA, USA). The beam was directed around the room on the floor and walls at random for 1 min, then the cat’s reaction was scored on the 0 to 2 scale.

#### 2.6.3. Ball

A ping-pong ball was presented to each cat. The investigator tossed the ball around the room and recorded the intensity of the cat’s interaction on the 0 to 2 scale.

#### 2.6.4. Social Interaction

During the social interaction task, all other enrichment options were removed and the cat was allowed to freely interact with the investigator who was seated on the floor. The investigator encouraged interaction by calling the cat’s name, patting her legs, and petting the cat if the cat chose to approach and interact. The investigator recorded the presence and intensity of the cat’s interaction on the 0 to 2 scale.

#### 2.6.5. Nail Conditioning Objects

In the final task, the investigator lured the cat back into his kennel with a treat and placed two nail conditioning objects into the cat’s kennel: A nail conditioning circle with a ball surrounding the conditioning material (Star Chaser Scratcher Cat Toy, Coastal Pet Products, Alliance, OH, USA) and an inclined nail conditioning cardboard box (KONG Naturals Incline Scratching Cat Toy, Kong Company, Golden, CO, USA; used without catnip) in the kennel with the cat. The investigator allowed the cat 1 min to scratch on either the circle or box. The investigator recorded the cat’s behavior, based on whether the cat scratched on one, both, or none of the nail conditioning objects.

### 2.7. Experimental Outcomes

The primary objective of these trials was to determine whether or not cats demonstrate a preference toward a specific type of enrichment. The secondary objective was to determine whether or not FIV status was associated with cat preferences.

### 2.8. Statistical Methods

Descriptive statistics were used to describe the percentages of cats showing each level of interest or preference for each enrichment type. For enrichment choice preference, a repeated-measures ordinal logistic regression model, PROG GENMOD (SAS Version 9.4, Cary, NC, USA) was used, with FIV status as a covariate for each of the enrichment types. The model allowed for the effect of day, order, and enrichment type. Linear contrasts were used to test specific effects.

## 3. Results

### 3.1. Animals

Twenty-three individually-housed cats completed this study. All 23 cats participated in six trials for each of the six enrichment options, with 138 trials (in total) per enrichment type. Mean weight of the cats (measured monthly) was 3.71 kg (range 3.4–4.56 kg). There were 17 FIV positive cats and six FIV negative cats. At the start of the study, FIV positive cats had been infected for 9–13 months. No adverse events were observed by the investigator or the animal technicians as a result of these experiments.

### 3.2. Preferences for Enrichment Type

#### 3.2.1. Brushes

In 109/138 trials (79.0%), cats showed interest in one or both brushes; in 29/138 trials (21.0%), cats showed no interest in either brush, preferring to explore the room or solicit attention from the investigator. When cats chose to interact with a brush, the glove brush was preferred in 44/109 trials (40.4%) while the human hairbrush was preferred in 23/109 trials (21.1%). Equal use of both brushes was found in 42/109 trials (38.5%).

#### 3.2.2. Laser, Ball, and Social Interaction

For each of these enrichment types, the number and percentage of the 138 trials that received each score are shown in Table 1. For each cat, the intensity scores were averaged over the six trials for each enrichment type, and the number of cats within each range of scores is shown in Table 2.

- There were no statistically significant differences present based on the order that each enrichment activity was presented (*p* = 0.16).
- The only statistically significant difference found in the analysis of preference was that the laser was preferred over the ball (*p* = 0.04) and over the social interaction enrichment option (*p* = 0.02).
- There were individual preferences for enrichment objects and a range of enrichment intensity scores for each cat.

#### 3.2.3. Nail Conditioning Objects

Of the two toenail conditioning objects, the majority of the 23 cats chose to scratch on the inclined box or they chose not to scratch at all during the time the toenail conditioning objects were offered. Of the 138 trials, cats chose to use one or both of the objects in 85 (61.6%). Of these 85 trials, cats chose the inclined box only in 69 (81.2%), the circle only in 3 (3.5%), and both the box and circle in 13 (15.3%). The cats in our study strongly preferred the inclined box to the circular nail conditioning object.

#### 3.2.4. FIV Status

No significant differences were found on any measure between FIV positive and FIV negative cats using a repeated-measures ordinal logistic regression (*p* = 0.57).

## 4. Discussion

The aim of this study was to determine laboratory cat preferences for enrichment items (brush, laser, ball, social interaction, and toenail conditioning objects), and to determine whether FIV status affected the cats’ preferences in these enrichments. We found that, in general, cats show a preference to be brushed, play with a laser, and scratch on an inclined-box nail conditioning object compared to play with a ball, interact socially with a familiar human, or scratch on a circular nail conditioning object. Further, cats appeared to have individual preferences for specific objects or interactions.

When the two different brush types (glove brush and hairbrush) were introduced to the cats for tactile stimulation, cats selected either the glove brush, or both brushes. Few cats selected the human hairbrush or the no-brush option. The overall choice by the cats for brush compared to no-brush, suggests that cats favor the opportunity for the tactile stimulation of grooming. Cats groom regularly by licking their fur with their tongues, and this behavior can lead to ingestion of hair and consequent hairball formation and vomiting [22]. Therefore, brushing to remove excess hairs can reduce hairball risk and promote positive interactions between caregivers and cat [1]. As the cats chose to participate in the brushing, the brushing sessions may be beneficial from both a health and welfare standpoint. Caretakers may also use the brushing period to inspect the cat for any other medical problems, including injuries not readily observable.

Using a comparison of the intensity of interaction scores as a surrogate for preference, we found that among the play objects, the laser was preferred over the ball and human interaction. The laser, a fast-moving, novel form of enrichment, may elicit predatory instincts of the cats, stimulating their natural species-specific hunting behaviors. Toys that move quickly seem to be especially attractive to cats, as such objects allow them to express the dual instincts to play and hunt after the play object [6]. It has been argued that the laser can be frustrating since the light can never be “caught” by the cats [6]. However, we did not observe signs consistent with “frustration” behavior, such as abandonment of laser-directed activity or redirected aggression. In general, the cats showed relatively little interest in the ball, although there were individual differences in cat interest. Compared to the laser, the ball was less kinetic and possibly less engaging to the cats. The cats also had more experience with the ball compared to other objects. In the facility, similar balls were occasionally thrown by caretakers during cleaning procedures, when cats were individually let out of the cages each day for exercise and social interaction. A ball was also left in the cages of the cats for enrichment by caretakers. This could make the ball less novel to the cats, in comparison to the laser. The effect of novelty, however, is inconsistent in enrichment studies, and context appears to be important. Studies in several species have shown that animals may preferentially approach [23] or avoid [24] novel objects, and that this effect may vary based on individual [25] and environmental factors [24]. Research in cats is lacking, however a study in tigers has found benefits of enrichment with novel toys [26] and in one study in dogs, novel toys were chosen over familiar toys in 76% of choice trials [23]. In addition, these findings must be interpreted in light of the fact that a comparison of intensity of interaction is not a true test of preference in the classical sense, where objects are compared simultaneously. As the enrichment options were presented serially, we used the intensity of interaction scores to evaluate the cats’ engagement, and a comparison between these scores to indicate preference. Future work with two-choice comparisons would be useful to further evaluate preferences.

Individual cats varied in their interest and the intensity of social interaction with the investigator. All cats scored higher on laser play compared to human social interaction. Social interaction with humans has been shown to positively impact the wellbeing of cats [6]. In addition, cats with human interactions have been shown to be easier to handle and more sociable [27]. Cat behavior can be impacted by the familiarity of the cat with the investigator [28]. During the pre-testing phase, the investigator noted that with increased familiarity, the cats selected to spend more time in close proximity.

The cats preferred the inclined box toenail conditioning object to the flat, circular nail conditioning object and, on the majority of trials, cats chose to use the opportunity to scratch. The cats tended to roll all over the inclined box and stretch across it. We suspect that the inclined nature of the box, allowing them to stretch and roll, made it preferable to the cats. Toenail conditioning objects allow cats to exhibit natural species-specific behaviors related to scent marking and claw-sharpening [29]. Fulfilling the biological need of nail conditioning is essential in any feline welfare plan. The observed preference for the inclined box over the flat circle may be related to the manner in which wild cats are known to scratch trees, by stretching up the surface of the tree and conditioning vertically [30].

The ranges in average intensity score show that cats differ individually in the intensity of their enrichment preferences, with mean intensity scores for each cat falling within a range from 0–2. This suggests that individual differences should be considered as a variable in an enrichment plan to enhance cat welfare, with preference testing performed on an individual basis from among enrichment items.

We did not see differences in enrichment preferences or intensity of interaction as a function of FIV status of the feline subjects. This study was conducted on cats with and without FIV; the range of infection time was 9–13 months. Since FIV is a chronic, progressive infection, these cats might not have been infected long enough to begin to show behavioral differences. Previous studies have shown that FIV cats do not show measurable cognitive motor decline until 12–18 or more months after infection. In fact, it has been shown that cats infected with FIV for two to three years have only small declines in cortical neuronal quantity [31]. Future studies should look at FIV positive cats with a longer infection time to determine if the length of infection has any impact on enrichment preferences.

With no significant differences found in behavioral responses between FIV positive and FIV negative cats, the overall enrichment preference trends identified in the present study may reasonably be extended to all individually housed laboratory cats. However, a limitation is the unequal FIV status categories (FIV+, *n* = 17; FIV−, *n* = 6), thus the generalizability of our data to a larger population of non-FIV infected research cats is unknown.

This study had several limitations. Care was taken to familiarize the cats with the investigator prior to the study as cats may show avoidance or inhibition of normal behavior in the presence of strangers [32]. It is not known if the findings of the present study can be generalized with regard to the familiarity of the cats with a person implementing the described enrichment strategies. In addition, cats were housed in individual cages as a requirement of the FIV study, whereas group housing is recommended for compatible cats without such restrictions [1]. Cats were housed in individual cages, with 5–6 cages within a room, and were tested individually in the rooms. However, in some instances when one focal animal was being tested, other cats, still in cages, became activated by the testing protocol, which could have affected the behavior of the focal animal. In addition, since a mandate of the laboratory animal facility was to provide some enrichment objects to all cats at all times, and a lack of enrichment would represent a welfare challenge, the cats were already exposed to many enrichment objects, on a rotating basis, within their cages. These objects included balls (Wiffle balls (The Wiffle Ball Inc., Shelton, CT, USA), jingle balls, golf balls, and extra small Best balls), or other objects such as crazy circles, shake rattle and roll toys, Whirlygigs, Nylabones, and PVC elbow pipes. Animal caregivers rotated the toy available to each cat weekly. The availability of a ball in the cats’ cages could have contributed to a lack of interest in the ping-pong ball provided to the cats during the trials, as the cats were already familiar with a ball, and the ball could have lost its novelty compared with other play object options; however, evidence of the effect of novelty on preference is inconclusive. The cats had also been previously exposed to the circle nail conditioning object. This familiarity could have contributed to the cats’ preference for the inclined box nail conditioning object. Anecdotally, since the end of this study and the recent availability of the inclined box nail conditioning object (in addition to the circle), caretakers have noted cat preference is consistent with the findings reported here. Further, the nutritional environment can be enriched through feeding techniques that extend the time the cat spends interacting with the food, such as food puzzles and delivery toys [11]. While a recent study suggested that food puzzles may not increase the overall daily activity of cats [33], they can increase their interaction with the food, more closely mimic natural behavior, and decrease aggressive, fearful behaviors, anxiety, and depression in housecats [11]. Further work could evaluate preferences for food puzzles in laboratory-housed cats as an enrichment form that stimulates natural feeding behaviors. Finally, as behavioral scoring is inherently subjective, a future refinement would include videotaping sessions and independent scoring of preferences from video.

## 5. Conclusions

Overall, both FIV positive and FIV negative cats showed a clear preference for specific enrichment items, reflected in either simultaneous presentation or higher ratings of intensity of interaction. These methods and findings may be used by laboratory animal facilities to select enrichment objects and interactions with laboratory cats. It has been shown that FIV is an effective model for studying HIV, in hopes that any vaccines developed for FIV can be extended to HIV. Therefore, it is critical to properly address the welfare needs of these models [34]. Long-term confinement, common in laboratory cats, is a welfare concern for animals, and enrichment is an integral component of their welfare. Enrichment items used in this study were commercially available at reasonable cost, could be cleaned effectively according to laboratory-facility standards, and were durable. Our finding of individual differences in enrichment preferences suggest that enrichment procedures should be adjusted based on behavioral traits of individual cats. Individually tailoring enrichment, based on preferences, would be a manageable task in laboratory animal settings.

## Figures and Tables

**Table 1:** Interest scores for laser, ball, and social interaction across the 138 trials. The number of trials at each score, and the percentage of the total trials is shown for each enrichment type. A score of 0 indicates no interest, while a score of 2 indicates high interest.

Enrichment Type	Score 0: *n* (%)	Score 1: *n* (%)	Score 2: *n* (%)
Laser	18 (13.0)	28 (20.3)	92 (66.7)
Ball	48 (34.8)	36 (26.1)	54 (39.1)
Social Interaction	54 (39.1)	37 (26.8)	47 (34.1)

**Table 2:** Average interest scores for laser, ball, and social interaction across the 23 cats. The number of cats with averages in each range are shown for each enrichment type. A score of 0 indicates no interest, while a score of 2 indicates high interest.

Enrichment Type	Average Score < 1: *n* (%)	Average Score 1.0–1.4: *n* (%)	Average Score 1.5–2: *n* (%)
Laser	4 (17.4)	4 (17.4)	15 (65.2) ^1^
Ball	11 (47.8)	2 (8.7)	10 (43.5)
Social Interaction	11 (47.8)	5 (21.7)	7 (30.4)

^1^ 11 of these cats scored 2 s in all 6 trials for the laser.
